# PHF8 and REST/NRSF co-occupy gene promoters to regulate proximal gene expression

**DOI:** 10.1038/srep05008

**Published:** 2014-05-23

**Authors:** Juan Wang, Xueqiu Lin, Su Wang, Chenfei Wang, Qixuan Wang, Xikun Duan, Peng Lu, Qian Wang, Chengyang Wang, X. Shirley Liu, Jinyan Huang

**Affiliations:** 1 Department of Bioinformatics, School of Life Science and Technology, Tongji University, Shanghai, 200092, China; 2 Fudan University, Shanghai Medical School, Department of Environmental Health; 3 Center for Functional Cancer Epigenetics, Dana-Farber Cancer Institute, Boston, MA 02215, USA; 4 Department of Biostatistics and Computational Biology, Dana-Farber Cancer Institute and Harvard School of Public Health, Boston, MA 02215, USA

## Abstract

Chromatin regulators play an important role in the development of human diseases. In this study, we focused on Plant Homeo Domain Finger protein 8 (PHF8), a chromatin regulator that has attracted special concern recently. PHF8 is a histone lysine demethylase ubiquitously expressed in nuclei. Mutations of PHF8 are associated with X-linked mental retardation. It usually functions as a transcriptional co-activator by associating with H3K4me3 and RNA polymerase II. We found that PHF8 may associate with another regulator, REST/NRSF, predominately at promoter regions via studying several published PHF8 chromatin immunoprecipitation-sequencing (ChIP-Seq) datasets. Our analysis suggested that PHF8 not only activates but may also repress gene expression.

Chromatin dynamics are affected by histone modifications. Chromatin regulators can alter histone modifications and thus are extensively linked to gene activation and repression^1^. Chromatin regulators are classified into the following three groups according to their functions: readers containing specific domains help to recognize and bind modified histone residues^2^; writers add post-translational modifications, such as methylation and acetylation^3^; and erasers remove post-translational modifications.

PHF8 is a JmjC domain-containing protein and erases repressive histone marks including H4K20me1 and H3K9me1/2^4^,^5^,^6^,^7^. It binds to H3K4me3, an active histone mark usually located at transcription start sites (TSSs)^8^,^9^, through its plant homeo-domain, and is thus recruited and enriched in gene promoters. Chromatin immunoprecipitation-sequencing (ChIP-seq) data from immortalized human HeLa cells show that about 72% of PHF8 binding sites are at promoters^5^. Also, PHF8 regulates the cell cycle biological process via removing H4K20me1 from the promoters of certain E2F1-regulated genes^5^. Interestingly, altering PHF8 levels in HeLa cells affects H4K20me1 methylation only in late G2/M and early G1 stages of the cell cycle, not globally^6^. PHF8 also binds to rRNA gene promoters and demethylates H3K9me2/1 to activate rRNA synthesis^10^,^11^, functioning as an activator^12^.

Nonsense or missense mutations in *PHF8* are associated with X-linked mental retardation (XLMR) and cleft lip/cleft palate^12^,^13^. ZNF711, another XLMR-associated protein, can recruit PHF8 to a subset of its target genes^4^. shRNA-mediated knockdown of either *ZNF711* or *PHF8* significantly decreases the expression of *ZNF711-PHF8* target genes, including *KDM5C*^4^. Previous study demonstrated that KDM5C, a histone demethylase that targets H3K4me2/3, is recruited by the repressor protein REST/NRSF and is also associated with XLMR^14^. Although these studies have revealed several important functions of PHF8, the regulatory functions of PHF8 remain poorly understood.

To investigate PHF8 regulatory functions, a motif-finding analysis for published PHF8 ChIP-seq data was conducted. The canonical REST motif was observed in PHF8 binding sites with high confidence. PHF8 targeting gene promoters were also bound by REST, which indicates that REST might interact with PHF8. Additionally, we detected the E2F1 motif in both PHF8 and REST ChIP-seq data. E2F1 is known to interact with PHF8^5^. According to our analysis results, PHF8, REST, and E2F1 shared significantly overlapped target genes and similar binding patterns, suggesting these three factors may form a trimeric complex. Moreover, differential expression analysis showed that PHF8 also has repressive function. Previous studies only reported PHF8 as an activator.

## Results

### PHF8 co-occupies gene promoters with E2F1 and REST

**Figure 1:**
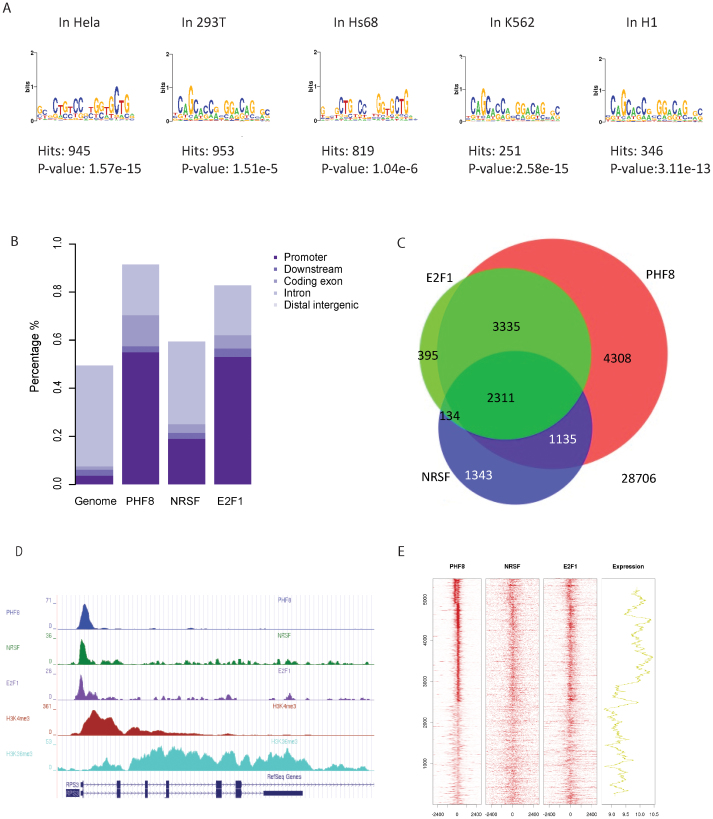
PHF8, REST, and E2F1 bind at genome-wide promoters. (A) Motif search of the highest-confidence PHF8 binding sites. Motifs in PHF8 binding sites surrounding gene promoters were searched. The REST motif was enriched in immortalized human cell lines HeLa, 293T, Hs68, and K562. (B) PHF8 and E2F1 are strongly enriched and REST is slightly enriched in gene promoters. Promoters, including 3 kb around the transcription start site and 5′UTR, were analyzed for PHF8, E2F1, and REST binding. Y-axis indicates the percentage of each region in the genome. (C) Overlap of PHF8, REST, and E2F1 target genes. Venn diagram shows the overlap of genes targeted by PHF8 (red), REST (blue) and E2F1 (green) in HelaS3 cells, with the number of genes indicated in each area. The significance of overlap (*p* < 1.9e-266) was calculated using hypogeometric distribution. (D) PHF8, REST, and E2F1 bind across the *RPS3* locus. The height of the profile indicates the number of sequenced reads, showed in y-axis. (E) PHF8, REST, and E2F1 co-occupy the genome. PHF8, REST, and E2F1 ChIP-seq profiles of 5,506 PHF8 target genes (y-axis) in a 6-kb window centered on gene transcription start sites. Expression box depicts expression trend using average sliding window with steps equaling 100. The far-right box shows the distribution of PHF8 up-regulated (red) and down-regulated (blue) genes.

Several PHF8 ChIP-seq datasets have recently been published in several immortalized human cell lines, including HelaS3, 293T, Hs68, K562 and H1^1^,^4^,^5^,^15^. MACS2 was performed on these data to detect PHF8 peaks (binding sites); then, MDSeqPos was applied to identify PHF8 binding motifs based on the top 1,000 confident peaks detected by MACS2^16^,^17^. REST canonical motif was significantly enriched in MDSeqPos results (Fig. 1A). We also identified an E2F1 motif in both PHF8 and REST ChIP-seq data (Fig. S1). As previous studies have already proved that PHF8 and E2F1 are co-factors, our results suggest that PHF8, E2F1 and REST are co-binding factors. GO-term analysis was performed on the overlapping target genes of PHF8 from HelaS3, K562 and H1 cell lines, and the significant pathway enrichment implicated a common regulatory mechanism of PHF8 across different cell lines (Table 1)^18^. We define genes that have peaks of certain factor within 3,000 base pairs around TSS (transcriptional start site) as target genes of this factor, for example, if within 3,000 base pairs of certain gene exists PHF8 peaks, this gene is one of PHF8 target genes.

Since bindings of PHF8 and E2F1 enriched in gene promoters, we deduced that these three factors maybe co-bind at gene promoters. In order to verify our assumption, we separately chose peaks for each factor with fold change greater than ten and calculated their overlaps. Here and in the remainder of this work, we processed data from HeLaS3 cell line because ChIP-Seq data of these three factors are all accessible in this cell line. Cis-regulatory Element Annotation System (CEAS)^19^ was applied to check the peak distribution of each factor on the genome. PHF8 and E2F1 were enriched at gene promoters, which is consistent with previous studies^15^,^20^, while REST was localized broadly across the genome (Fig. 1B). Then, we checked the co-locations of these three factors. As expected, peaks overlap was not significant throughout the genome since PHF8 and E2F1 bindings were only enriched in promoters. (Supplementary Fig. S2A). However, the functional peaks (peaks located in gene promoters) of PHF8, REST, and E2F1 are highly overlapped (Supplementary Fig. S2B). Also, they shared a large portion of their target genes (Fig. 1C), which implied these three factors tend to target similar group of genes by binding to gene promoters.

If PHF8 directs REST and E2F1 to its target genes, they should co-occupy PHF8 target gene promoters. As it is not efficient to study genes with undetectable expression, we only focused on the expressed genes to check the binding status of these three factors. Occupancies of PHF8, REST and E2F1 across the 5,506 expressed PHF8 target genes revealed that these three factors co-occupied most of gene promoters, such as *RPS3* (Fig. 1D and E), though some of the occupancies are relatively weak. In comparison to the expression of all the Refseq genes, the average expression of the 5,506 PHF8 target genes was significantly higher (*p* < 2.2e-16 by Student's t-Test) (Supplementary Fig. S3). We further investigated whether the strength of PHF8, REST, and E2F1 binding sites would affect target genes expression. The gene expression values have been smoothed to make the trend clearer and easier to be noticed by using an average sliding window. Strong factor signals corresponded with high gene expression levels and vice versa. These results indicate that these three factors associate with each other at expressed PHF8 target genes promoters to regulate their expression (Fig. 1E).

**Figure 2:**
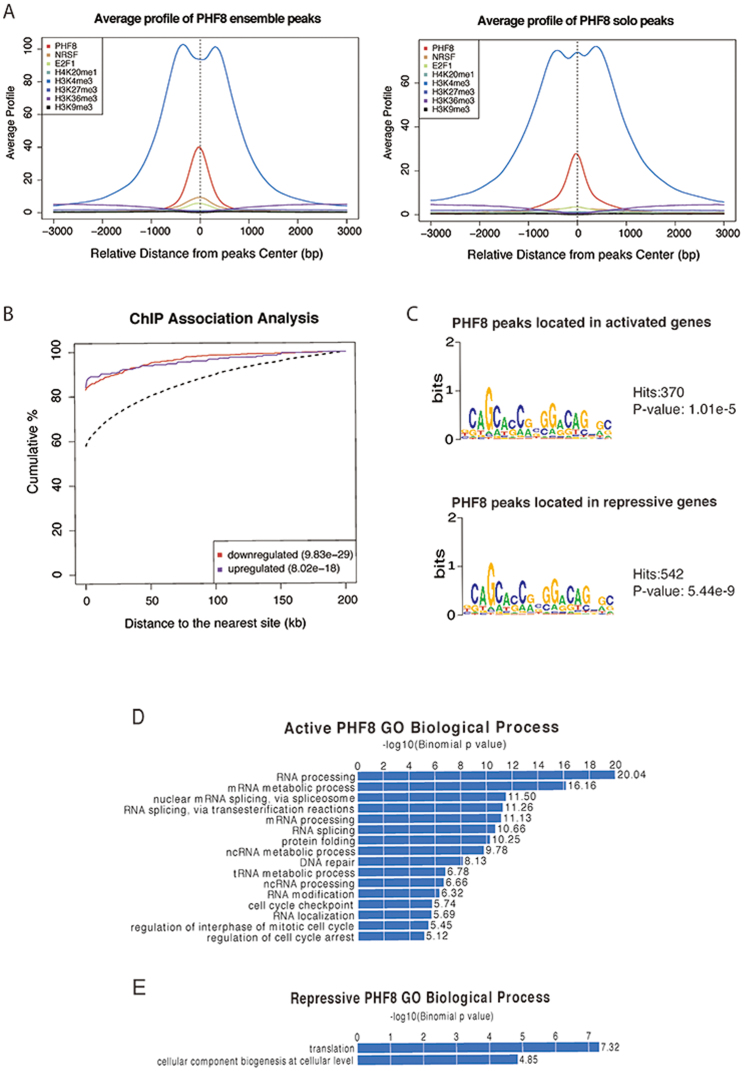
PHF8 activates and represses gene expression. (A) Average binding signal profile around PHF8 ensemble and solo peaks center. PHF8, REST, E2F1, H4K20me1, H3K4me3, H3K27me3, H3K36me3, and H3K9me3 average binding signal profiles for PHF8 ensemble and solo peaks. (B) Cumulative distribution of PHF8 binding sites within certain distances of transcription start sites. ChIP association analysis of PHF8 binding at transcription start sites. Red line depicts up-regulated genes after *PHF8* knockdown; blue line depicts down-regulated genes; and dashed line depicts all genes. (C) Motif analysis in PHF8-activated or -repressed genes. Motif finding was performed in peaks surrounding the top 1000 PHF8-activated and -repressed targets. (D) Processes associated with activated PHF8. GREAT analysis identified biological processes associated with activated PHF8 peaks. (E) Processes associated with repressed PHF8. GREAT analysis identified biological processes associated with repressed PHF8 peaks.

To investigate the chromatin state differences between sites bound by PHF8 only and PHF8-REST co-binding, PHF8 peaks were divided into two groups according to whether the peaks are co-occupied by REST or not. We detected 2,780 PHF8 peaks with REST co-binding (PHF8 ensemble peaks) and 10,562 peaks without REST co-binding (PHF8 solo peaks). To make the average binding signal profiles comparable between these two groups, 2,780 peaks were randomly selected from the PHF8 solo peaks. The average binding signal profiles of H3K4me3, PHF8, and E2F1 on the PHF8 solo and ensemble peaks showed that their binding would be stronger with REST co-binding (Fig. 2A), indicating that REST may strengthened their binding. Closer examination of the H3K4me3 profile revealed a small central peak only in the PHF8 solo peaks. This may imply that with REST, PHF8 fully occupy the peak center without H3K4me3. However, without REST, H3K4me3 co-occupies the peak center with PHF8. Other histone marks did not significantly change.

### PHF8 upregulates and downregulates gene expression

PHF8 is claimed to be an activator^4^,^5^,^6^,^7^,^15^. However, according to the PHF8 regulatory function analysis, it may also have the potential to act as a repressor. To fully identify PHF8 regulatory functions, we defined differentially expressed genes upon *PHF8* knockdown or over-expression. We used GEO dataset GSE20563^15^ to identify the most differentially expressed genes (adjusted p-value < 0.001, by Student's t-test) after *PHF8* knockdown. The expression of 261 genes decreased after *PHF8* knockdown, while 530 genes increased; we defined these groups as PHF8 up-regulated genes and PHF8 down-regulated genes, respectively. The distributions of PHF8 up- and down-regulated genes on all expressed PHF8 target genes do not show strong pattern, that suggest PHF8 binding strength has no influence on the gene differential expressions (Fig. 1E).

Though the genes were up- or down-regulated under the PHF8 knockdown condition, it is uncertain whether these two groups of genes are truly regulated by PHF8. As we know, transcription factors usually regulate genes by binding to their promoters. Thus the distance between PHF8 binding sites and differentially expressed genes, to a great extent, reflects the regulatory potential (potential is higher when PHF8 binding sites are closer to the TSS). For both PHF8 down-regulated and up-regulated genes, distances between PHF8 binding sites are significantly smaller than those background genes (down-regulated, *p* = 9.83e-29 by one-sided Kolmogorov-Smirnov test; up-regulated genes, *p* = 8.02e-18 by one-sided Kolmogorov-Smirnov test) (Fig. 2B), indicating that PHF8 not only up-regulates but also down-regulates gene expression.

In order to find out whether REST plays a role in modulating PHF8 regulated genes, genes with both high differential expression and high regulatory potential are chosen, including 1,000 up-regulated (repressive) and 1,000 down-regulated (active) genes. In comparison to all genes, active (*p* = 9.29e-155 by one-sided Kolmogorov-Smirnov test) and repressive (*p* = 1.48e-193 by one-sided Kolmogorov-Smirnov test) gene expression were significantly regulated by PHF8 (Supplementary Fig. S4). When performing motif analysis for PHF8 peaks located in promoters of both repressive and active genes, REST motif was detected with high confidence (Fig. 2B and C). Comparing to the motif detected from random located peaks, REST motif possessed more significant *p*-values and higher hits (Supplementary Fig. S5). These results indicate that PHF8 may co-regulate gene expression with REST.

### Functional analysis of differentially expressed genes

Functional analysis of PHF8 differentially expressed genes gives us a clear view of biological pathways that PHF8 involves in. Both DAVID and GREAT were used to analyze the functions of PHF8 active and repressive genes^18^,^21^. GREAT analysis suggested that PHF8 active genes were significantly enriched in biological processes including RNA processing, DNA repair, and cell cycle checkpoints (Fig. 2D); PHF8 repressive genes were barely enriched for any biological processes (Fig. 2E). DAVID analysis provided concordant results (Table 2). According to this functional analysis, PHF8 and REST co-bounding activated genes have functions in RNA processing, DNA repair, cell cycle checkpoint biological process and etc.

In eukaryotes, differential gene expression can be affected by controlling the level of RNA processing and transport. Therefore the RNA processing process might relate to the regulation of development. DNA repairing is related to the prevention of tumor. PHF8 binds to a subset of E2F1 regulated genes, and functions in the regulation of cell cycle^5^.

## Discussion

This analysis revealed that REST and E2F1 associate with PHF8 at a subset of PHF8 target gene promoters to influence gene expression. Further, PHF8 has both active and repressive functions in influencing genes expression. These findings reveal a new aspect of studying the function of PHF8 and REST. Although we identified an association between PHF8 and REST at gene promoters, the specific mechanism of how they work together remains uncovered.

These results indicate that PHF8-activated genes are co-bound by REST and function in RNA processing, DNA repair, and regulation of cell cycle checkpoints. RNA processing and transport control can differentially affect gene expression, providing a potential mechanism to regulate genes expression. DNA damaging can prompt unregulated cell division and contribute to the development of multiple diseases, and this process is prevented by DNA repairing. Moreover, PHF8 binds to a subset of E2F1-regulated genes that influence cell cycle regulation^5^. These results suggested that PHF8 might play a central role in several aspects of cellular function.

Mutations in *PHF8*, in addition to numerous other genes, cause XLMR. PHF8 also interacts with ZNF711 to affect expression of XLMR gene *KDM5C*^22^. KDM5C binds H3K9me3 via its N-terminal PHD domain to act as an H3K4me3 demethylase and interacts with the REST motif to remove tri-methyl modifications on H3K4me3^14^. Together, these proteins are linked by a pathway that suppresses XLMR^4^, suggesting KDM5C may also interact with PHF8. KDM5C may bridge PHF8 and REST functions in XLMR, providing new insight for the disease mechanisms of XLMR. The carboxy-terminus of REST recruits a large complex through co-represssor CoREST, including H3K4 demethylase LSD1^23^ and the H3K9 histone methyltransferase G9a^24^. Both LSD1 and G9a mediate modifications associated with gene silencing^25^.

## Methods

### ChIP-seq data analysis

GSE20725 and GSE22478 are PHF8 and E2F1, respectively, ChIP-seq datasets from GEO (http://www.ncbi.nlm.nih.gov/geo/)^5^,^15^; REST ChIP-seq data are from ENCODE (http://encodeproject.org/ENCODE/). Peak calling was performed using MACS2, a new version of MACS^26^ with a cutoff of *q* = 0.01. 14,402 and 11,159 peaks are called in PHF8 and E2F1 ChIP-seq data set, respectively; 28,689 peaks are called in REST ChIP-seq data set. Genome-wide peak distributions were generated with CEAS^19^.

### Expression data analysis

Gene expression data were derived from GEO (GSE20563)^15^ and analysis procedures were performed using R (version 2.13.1). Package Agi4*44PreProcess was used for primary processing; Agilent Feature Extraction software was used for image analysis; and Chip hgug4112a was used for annotation^27^. For background correction, we used the *background correct* function of the limma package with the option of *half*, which is designed to produce positive-corrected intensities. After background correction, data were normalized between arrays using the *normalize between arrays* function of limma with the option of *quantile*^28^.

### Motif finding

DNA motifs were identified using MDSeqpos (X. Shirley Liu laboratory). All binding sites were trimmed or extended to 600 bp and centered at the summit of the peak region identified by MACS2. MDSeqpos searched both *de novo* and known DNA motifs from the database to examine motif enrichment among peak regions.

### Up-regulation or down-regulation prediction

A tool developed by Hyunjin Gene Shin (X. Shirley Liu laboratory) was used to investigate whether a transcription factor or a chromatin regulator is a repressor or an activator. This tool draws the cumulative distribution for each gene set and tests if each distribution is identical using the one-sided Kolmogorov-Smirnov test.

## Author Contributions

J.W. and J.H. analyzed the results and wrote the manuscript; J.W., X.Q.L., S.W. and C.F.W. prepared the figures. Q.X.W., X.K.D., C.Y.W., P.L., Q.W. and S.X.L. provided key suggestions. All authors reviewed the manuscript.

## Supplementary Material

Supplementary Information SUPPLEMENTARY

## Figures and Tables

**Table 1:** DAVID analysis of PHF8 overlapping target genes from H1, Hela and K562

Gene group	Term	Count	*p-value*	FDR
**PHF8 overlapping target genes from H1, HelaS3 and K562.**	Cellular metabolic process	1269	1.9E-45	7.7E-42
	Cellular protein metabolic process	521	2.5E-24	1.5E-21
	RNA metabolic process	245	4.8E-20	2.5E-17
	Cellular catabolic process	238	6.2E-13	1.5E-10
	Cell cycle	183	1.2E-10	1.9E-8
	RNA biosynthetic process	82	2.4E-8	2.8E-6

**Table 2:** DAVID analysis of PHF8 binding sites with active or repressive functions

Gene group	Term	Count	*p-value*	FDR
**PHF8-activated targets**	Macromolecule metabolic process	427	6.56E-24	1.17E-20
	Gene expression	281	1.12E-21	1.99E-18
	Cellular process	682	2.70E-16	4.00E-13
	Transcription	195	1.34E-13	2.38E-10
	Chromatin organization	57	1.84E-07	3.27E-04
	RNA splicing	38	1.38E-06	2.45E-03
	Cell cycle process	57	2.12E-05	3.78E-03
