# Supplementary material for: PHF8 and REST/NRSF co-occupy gene promoters to regulate proximal gene expression
Source: Sci Rep. 2014 May 23;4:5008. doi: 10.1038/srep05008 (PMC4031473; doi:10.1038/srep05008)
Supplement: Supplementary Information — SUPPLEMENTARY [file srep05008-s1.doc]

**PHF8 and REST/NRSF co-occupy gene promoters to regulate proximal gene expression**

Juan Wang^1^, Xueqiu Lin^1^, Su Wang^1^, Chenfei Wang^1^, Qixuan Wang^1^, Xikun Duan^1^, Qian Wang^1^, Chengyang Wang^1^, X. Shirley Liu^2, 3^, Jinyan Huang^1^

1. Department of Bioinformatics, School of Life Science and Technology, Tongji University, Shanghai, 200092, China;

2. Center for Functional Cancer Epigenetics, Dana-Farber Cancer Institute, Boston, MA 02215, USA

3. Department of Biostatistics and Computational Biology, Dana-Farber Cancer Institute and Harvard School of Public Health, Boston, MA 02215, USA.

Correspondence: Jinyan Huang, Email: [jyhuang@tongji.edu.cn](mailto:jhuang@tongji.edu.cn).

**Figure S1, E2F1 motif is significant in top 1,000 NRSF peaks in Hela.**

**
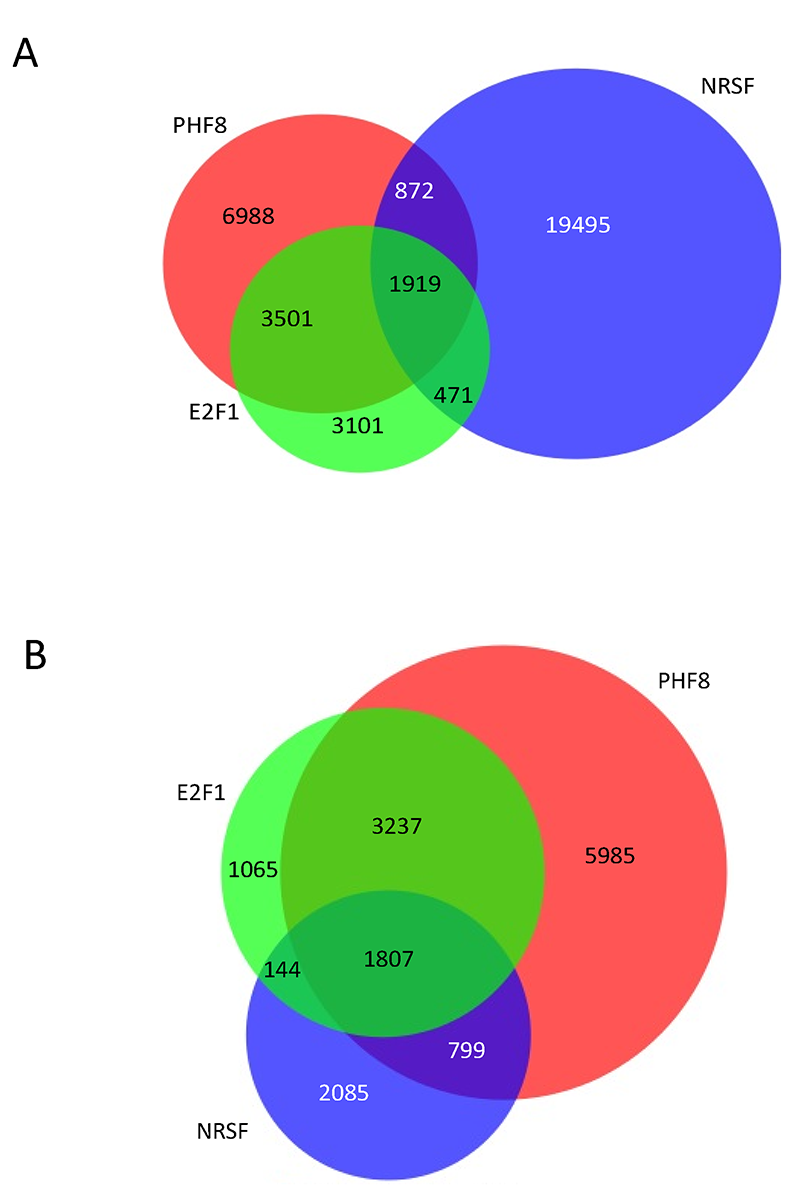
**

**Figure S2, Overlap of the three factors binding sites in genome and in gene promoters.**


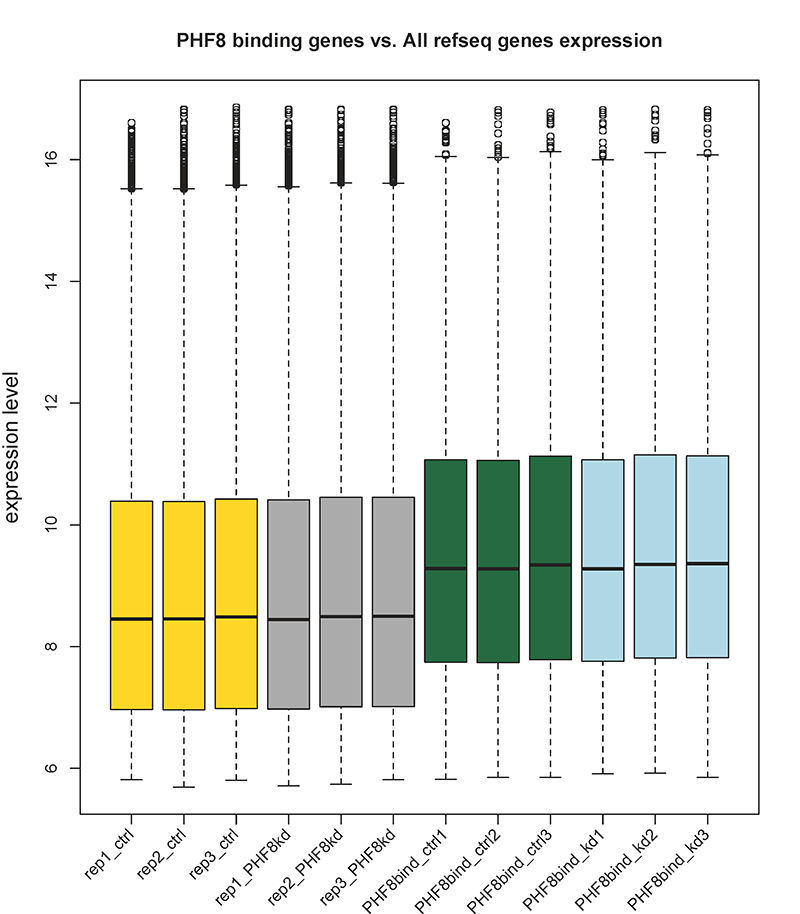


**Figure S3, Comparison between PHF8 targeted genes and all the refseq genes expression.** Bars in gold represent all the refseq genes expression in wild type Hela cells, while bars in grey are Hela cells with PHF8 knockdown. Bars in dark green and light blue represent PHF8 targeted genes in wild type and PHF8 knockdown respectively. Each of them has three replicates.


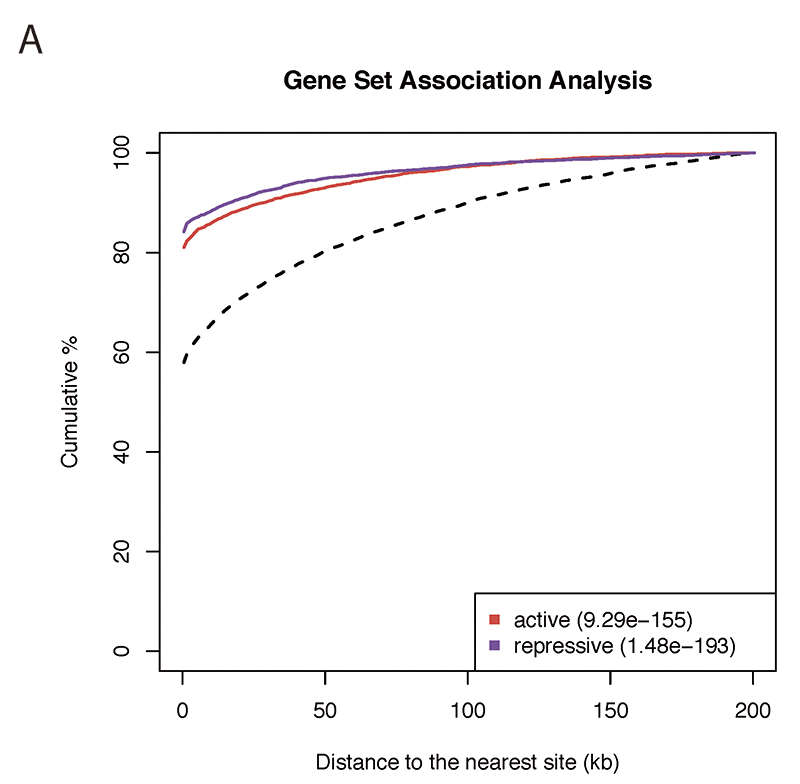


**Figure S4, The cumulative line of PHF8 binding sites within certain distances of gene TSSs.** Choose genes with both high differential expression and high regulatory potential.


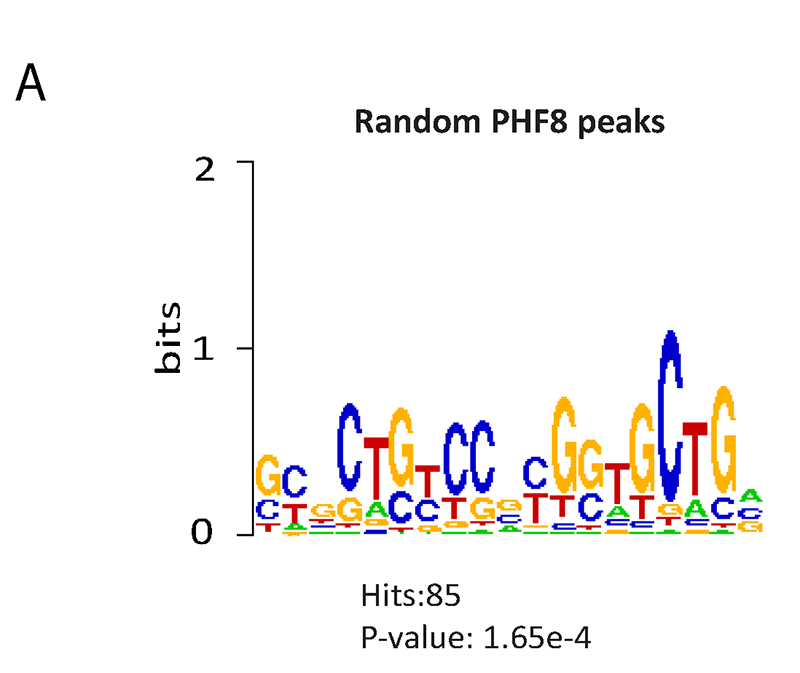


**Figure S5, Motif finding in random PHF8 peaks.**

**Table S1.** DAVID analysis of PHF8 binding sites with strong and weak signals.

| **Gene group** | **Term** | **Count** | ***p-value*** | **FDR** |
| --- | --- | --- | --- | --- |
| **Strong signal PHF8 targets**  **Weak signal PHF8 targets** | Cellular metabolic process  Cellular process  RNA splicing  Translation  Gene expression  Cell cycle  Cellular metabolic process  Cellular process  Cell cycle  Mitotic cell cycle  Regulation of cell cycle | 1256  1708  107  95  537  169  1464  2114  230  127  115 | 1.21E-59  8.51E-47  1.60E-23  2.68E-12  3.22E-12  1.00E-09  1.30E-34  1.72E-33  2.24E-16  3.00E-14  2.18E-13 | 2.24E-56  1.57E-43  2.96E-20  4.96E-09  5.96E-09  1.85E-06  2.47E-31  3.26E-30  4.22E-13  5.69E-11  4.13E-10 |
